# Supplementary material for: Structurally Different Yet Functionally Similar: Aptamers Specific for the Ebola Virus Soluble Glycoprotein and GP1,2 and Their Application in Electrochemical Sensing
Source: Int J Mol Sci. 2023 Feb 27;24(5):4627. doi: 10.3390/ijms24054627 (PMC10003157; doi:10.3390/ijms24054627)
Supplement: Supplementary file 1 [file ijms-24-04627-s001.zip › ijms-2167280-supplementary.pdf]

Supplemental Materials

Structurally different yet functionally similar: Aptamers specific for the Ebola virus soluble glycoprotein and GP1,2 and their application in electrochemical sensing.

Soma Banerjee, Mahsa Askary Hemmat, Shambhavi Shubham, Agnivo Gosai, Sivaranjani Devarakonda, Nianyu Jiang, Charith Geekiyanage, Jacob Dillard, Wendy Maury, Pranav Shrotriya, Monica H. Lamm, and Marit Nilsen-Hamilton

Table of contents:

Sequences of proteins used in this study

Tables

Table S1: Comparison of available aptamers
Table S2: Comparison of sensors for Ebola virus

Figures

Figure S1: Specificity of oligonucleotide 6012
Figure S2: Titrations of four antibodies to establish EC50 for sGP
Figure S3: Test for competition between EBOV sGP binding aptamers and four EBOV GP antibodies
Figure S4: 2D and 3D structure predictions of 6011, 6012, 70SGP2A, and 39SGP1A aptamers
Figure S5: Overlaid models for aptamer docking to sGP compared with antibody epitopes
Figure S6: Stability of oligonucleotide 6011 at high temperatures
Figure S7: Binding isotherms for oligonucleotides 6020 and 6022
Figure S8: Thermal stability of 39SGP1A as RNA and 2'FY-RNA
Figure S9: Electrochemical sensing Nyquist plots for Fig. 7
Figure S10: Schematic of electrochemical sensor

Sequences of proteins used in this study

A. Sequences of sGP used for experimental testing and molecular modeling

The sequence of sGP secreted by transfected HEK293T cells and purified as described in Materials and Methods is shown in black letters. The amino acids residues that are included in the structures deposited in the pdb database (5KEM) are shown as bold and red letters:

MGVTGILQLPRDRFKRTSFFLWVIILFQRTFSIPLGVIHNSTLQVSDVDKLV**CRDKLSSTNQLRSVGLNL**
EGNGVATDVPSATKRWGFRSGVPPKVVNYEAGEWAENCYNLEIKKPDGSECLPAAPDGIRGFPRCR
YVHKVSGTGPCAGDFAFHKEGAFFLYDRLASTVIYRGTTFAEGVVAFLILPQAKKDFFSSHPLREPVN
ATEDPSSGYYSTTIRYQATGFGTNETEYLFEVDNLTYVQLESRFTPQFLLQLNETIYTSGKRSNTTGKL
IWKVNPEIDTTIGEWAFWETKKTSLEKFAVKSCLSQLYQTEPKTSVVRGSLVPRGSPHHHHHH

B. Sequences of GP1,2 used for experimental testing and GP1 used for molecular modeling

The sequence of the GP1,2 fusion protein purchased from Sino Biological is shown in black letters. The amino acids residues that are included in the structure of GP1 deposited in the pdb database (5KEN) are shown as bold and red letters:

IPLGVIHNSTLQVSDVDKL**CRDKLSSTNQLRSVGLNLEGNGVATDVPSVTKRWGFRSGVPPKVVNYE**
AGEWAENCYNLEIKKPDGSECLPAAPDGIRGFPRCRYVHKVSGTGPCAGDFAFHKEGAFFLYDRLA
STVIYRGTTFAEGWAFLILPQAKKDFFSSHPLREPVNATEDPSSGYYSTTIRYQATGFGTNETEYLFEV
DNLTYVQLESRFTPQFLLQLNETIYASGKRSNTTGKLIWKVNPEIDTTIGEWAFWETKKNLTRKIRSEE
LSFTAVSNGPKNISGQSPARTSSDPETNTTNEDHKIMASENSSAMVQVHSQGRKAAVSHLTTLATISTS
PQPPTTKTGPDNSTHNTPVYKLDISEATQVGQHHRRADNDSTASDTPPATTAAGPLKAENTNTSKSAD
SLDLATTTSPQNYSETAGNNNTHHQDTGEESASSGKLGLITNTIAGVAGLITGGRRTRREVIVNAQPKC
NPNLHYWTTQDEGAAIGLAWIPYFGPAAEGIYTEGLMHNQDGLICGLRQLANETTQALQLFLRATTELR
TFSILNRKAIDFLLQRWGGTCHILGPDCCIEPHDWTKNITDKIDQIIHDFVDKTLPDQGDNDNWWTGWR
QAHHHHHHHHHH

Tables

Table S1. Comparison of aptamer characteristics.

Characteristic features	Aptamer 6011 and 70SGP2A reported here	**Previously reported aptamers for Ebola virus**	
		**39SGP1A [1]**	**GP-B03, D01 and G-02 [2]**
Aptamer type	ssDNA	2'FY RNA	ssDNA
Stability in absence of serum	Stable within the experimental time fame of 60 days	Stable within the experimental time frame of 60 days	Not determined
Stability in 50% serum	Half-life = 32 d	Stable over the 80-day experimental time frame	Not determined
Affinity for Ebola GP1,2	K_d = 48 and 58 nM	K_d =104 nM	K_d = 4.1 to 14 nM
Affinity for Ebola sGP	K_d = 8.5 and 27 nM	K_d 13 nM	Not determined
Cross-strain recognition	Recognizes EBOV and Sudan	Recognizes EBOV and Sudan	Not determined
Functional in a sensor	Yes	Not determined	Not determined
Cost effectiveness	Low cost	Higher cost due to 2'FY modification	Low cost
LOD on sensor	150 pM		61 pM*

Legend: The aptamers reported to be selected in this work are compared with previously characteristics of aptamers selected against sGP (39SGP1A) or GP1,2 aptamers (GP-B03, D01, and G02). *The estimate of LOD was reported as 4.2 ng/mL [2]. Our calculation for the Molar value of the LOD was based on the molecular weight of the GP1,2 preparation that we used in this work and the expectation that it is a monomer as the authors did not identify a molecular domain (such as a foldon) in the protein that holds the monomers together as a trimer.

Table S2. Comparison of sensors for Ebola virus.

Sl. No.	Reference	Method	Transducer / Target	Detection limit	Time
1	[3]	Chromatography / LFA	Magnetic nanoparticle based nanozyme / Zaire GP	2 nM in diluted serum	30 mins
2	[4]	Chromatography	Nitrocellulose membrane / Zaire GP	2.7 nM in blood, plasma	
3	[5]	Optical / single particle interferometry	Antibody on silicon substrate / pseudotyped virus particles	5×10^3 pfu/ml blood, plasma	2 hours
3	[6]	Opto-fluidic nanoplasmic	Antibody/pseudotyped virus particles	106 pfu/ml in PBS	90 mins
4	[7]	Opto-fluidic	Molecular beacon, fluorescence / Ebola RNA	0.2 pfu/ml in water	3-10 mins
5	[8]	Electrochemical FET	Antibody on alumina/graphene oxide / Zaire GP	18 pM in 1% serum	Few seconds
6	ReEBOV™	Chromatography / LFA	VP40	11.4 nM in blood	15 mins for qualitative
7	[9]	Molecular diagnostic method	Reverse transcriptase loop-mediated isothermal amplification (RT-LAMP) of the glycoprotein gene	2.8 × 102 plaque-forming units (PFU)/test in lyophilized samples	40 mins
8	[10]	Optical (plasmonic)	Antibody on 3D gold nanoantenna / EBOV sGP	10.7 pg/ml in human plasma (1:12 000 dilution)	
9	[11]	Electrochemical FET	Antibody on reduced graphene oxide / inactivated EBOV particles	12 pg/ml in 10% human serum	
10	This work	Electrochemical impedance	Aptamer on nanoporous alumina / sGP and virus particles	150 pM in 10% human serum	2-3 hours for quantitative / 30 mins for qualitative

Legend: The results of this work are compared with other reported sensors for detecting Ebola infection. Most reported sensors either detect GP or virus particle, whereas the current work detects sGP, which is found in abundance in the blood of an infected individual. For a qualitative (YES/NO) output, the current sensing mechanism can produce result in 30 minutes (3 EIS scans of a sample) whereas a quantitative determination could be made in 3 hours (EIS scans of six different dilutions of the sample to compare against a standard calibration curve). The sensor reported in this work can also detect GP expressed on the surface of virus particles.

Figures

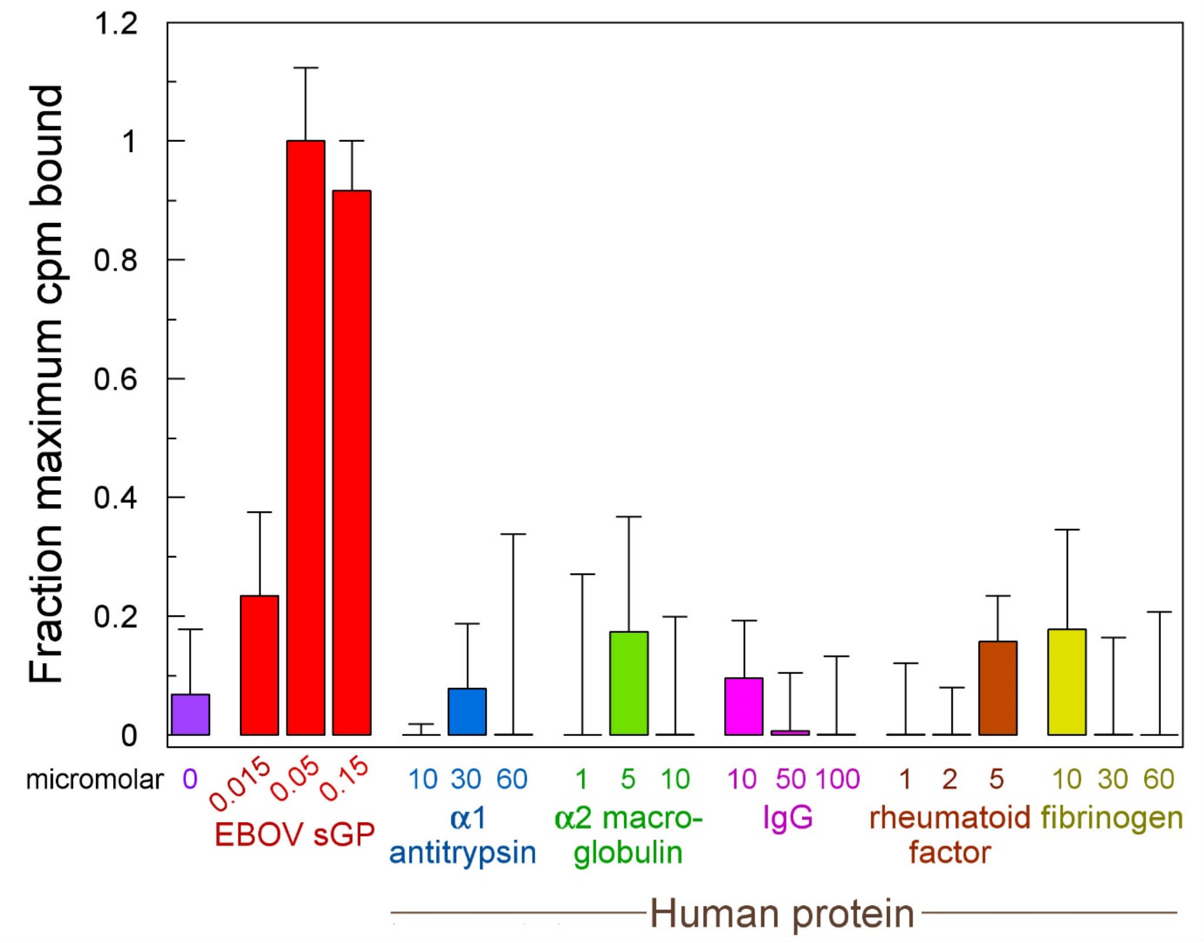

Legend: Radiolabeled oligonucleotide 6012 was incubated with the identified concentrations of each protein and the amount bound to protein was determined by filter capture for each condition. Cpm bound for duplicates for each condition were averaged and the average of blanks (no protein) were subtracted from values of samples with protein. The average cpm (-blank) bound for each condition was normalized to the average cpm (-blank) bound by 0.15 μM sGP. In each titration, the concentrations of the proteins are for the quaternary structures, which are the sGP homodimer, IgG, heterotetramer and monomers for the other listed proteins. All binding assays were performed in PBS at 24 °C. Standard deviations were calculated as described in materials and methods to include the error in the blank when that was subtracted.

Figure S1. Specificity of oligonucleotide 6012.

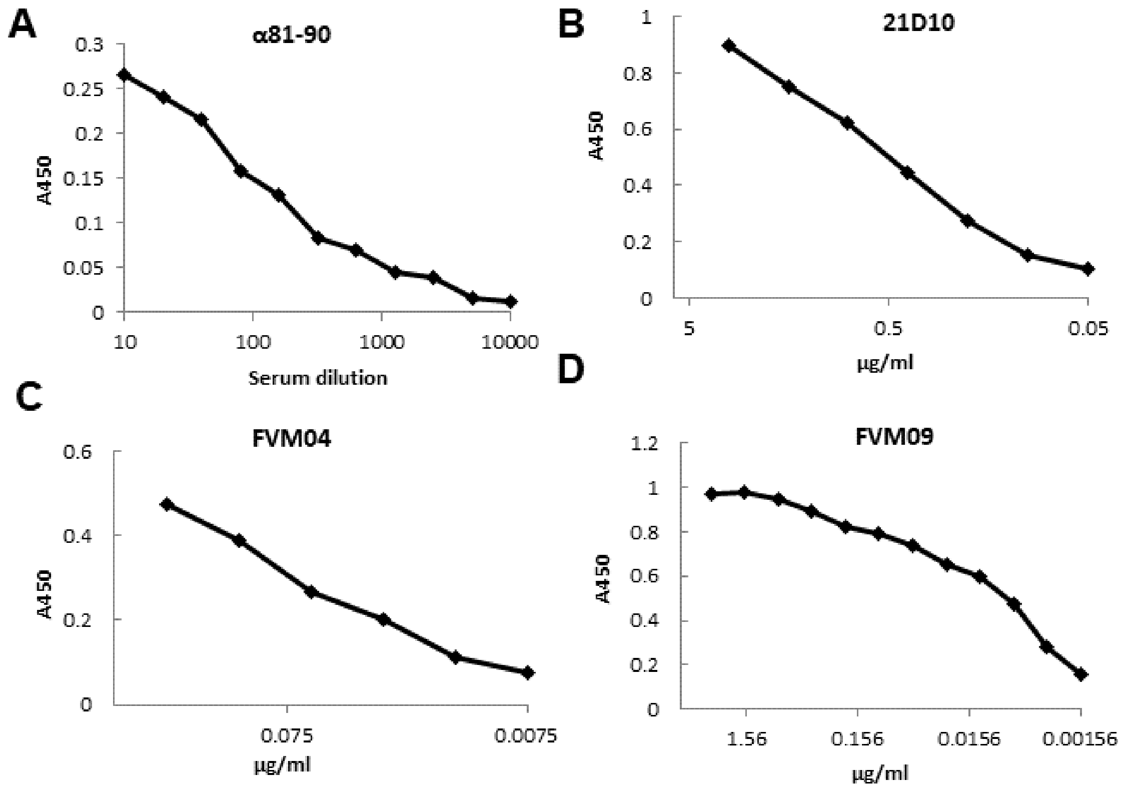

Legend: The binding to sGP of serial dilutions of rabbit antisera against EBOV GP amino acids 83-96 (A) or α-EBOV GP1 monoclonal antibodies (mAb) (B-D) to sGP. The ELISA assay was performed as described in Materials and Methods with the identified antibodies at the concentrations noted in the figure legends. mAb 21D10 binds to EBOV GP residues 81-90, FVM09 binds to residues 286-290 and FVM04 binds to a conformational epitope. The binding curves were used to determine the ~EC_{50} concentration.

Figure S2. Titrations of four antibodies to establish EC50 for sGP.

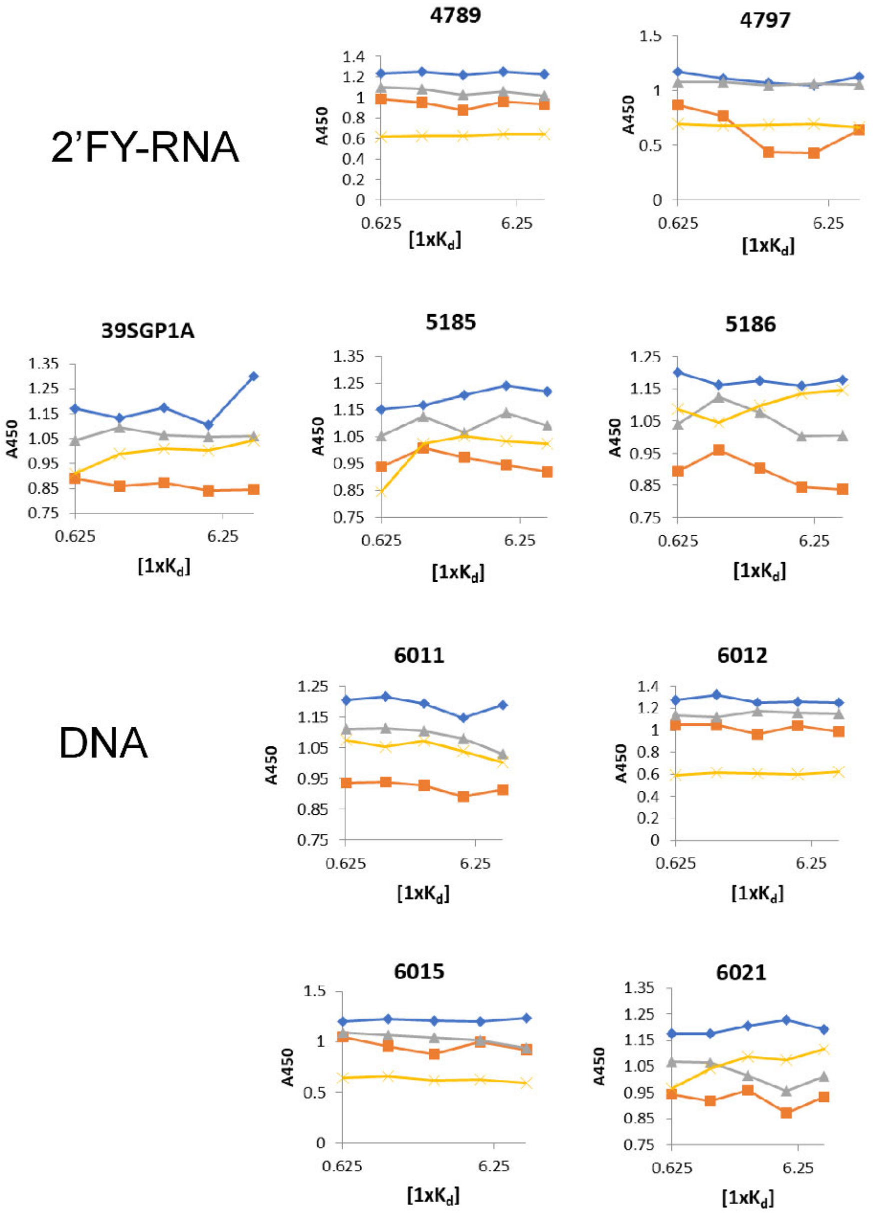

Legend: Four EBOV antibodies were tested, each at their IC50 concentration (Fig. S2), for competition with a series of oligonucleotides that bind sGP by ELISA. The antibodies were: 21D10 (gray), FVM04 (blue) or FVM09 (orange) or rabbit antisera (α85-98) (yellow). DNA aptamers were tested that were identified in the SELEX population described in this work (6011, 6012, 6015 and 6021). 2’FY-RNA aptamers identified in previous SELEX experiments [1]. The concentrations used for each oligonucleotide were 0.625-fold to 6.25-fold the approximated K_d for sGP binding. The ~Kd’s used for this study were: 4789 (140 nM), 4797 (500 nM), 39sGP1A (50 nM), 5185 (50 nM), 5186 (500 nM), 6011 (100 nM), 6012 (90 nM), 6015 (39 nM), 6021 (20 nM).

Figure S3. Test for competition between EBOV sGP binding aptamers and four EBOV GP antibodies.

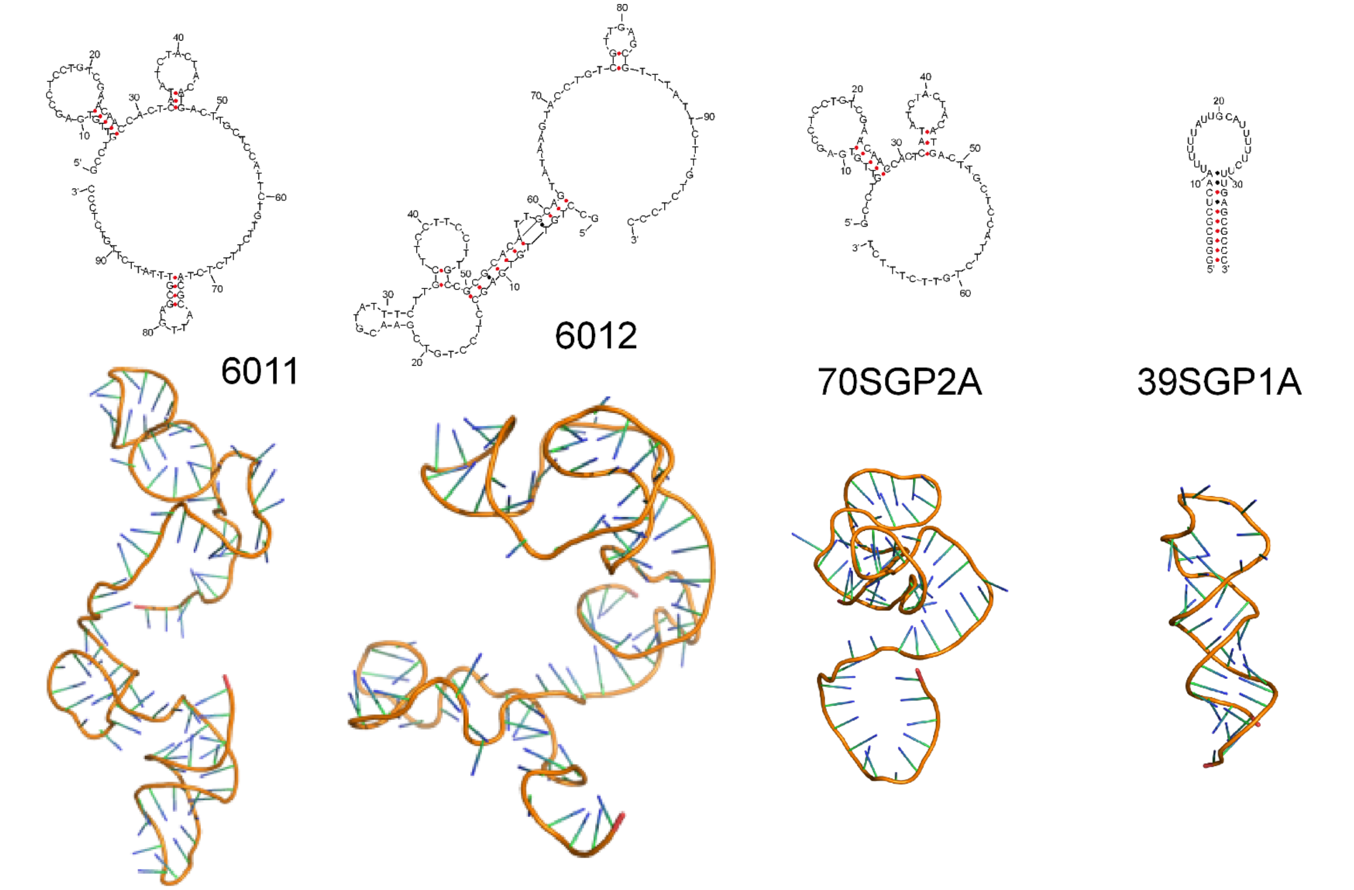

6011:GCCTGTTGTGAGCCTCC
TGTCGAACAACCACTCATATC
TACTACATGACTTGCTCCATTC
TGTTCTTTCTCTACGCATTGAG
CGTTTATTCTTGTCTCCC

6012:GCCTGTTGTGAGCCTC
CTGTCGAACGTATTTCTTGCT
TCCTTCCTTGCCGCGCACATT
GCAGTATAAGTACCTGTCGTT
GAGCGTTTATTCTTGTCTCCC

70SGP2A:GCCTGTTGTGAGC
CTCCTGTCGAACAACCACTC
ATATCTACTACATGACTTGCT
CCATTCTGTTCTTTCT

39SGP1A:GGGCGCUCA
AUUUUUUAUUGCAUUU
UUCUUUGAGCGCCC

Legend: Predicted two- and three-dimensional structures for the 6011, 6012, 70SGP2A, and 39SGP1A aptamers. The primary sequence is shown in the bottom row. The 2D structures were predicted using the UNAFold web server (http://www.unafold.org/) [12]. The 3D structures were predicted using the 3dRNA/DNA web server (http://biophy.hust.edu.cn/new/3dRNA) [13; 14].

Figure S4. 2D and 3D structure predictions of 6011, 6012, 70SGP2A, and 39SGP1A aptamers.

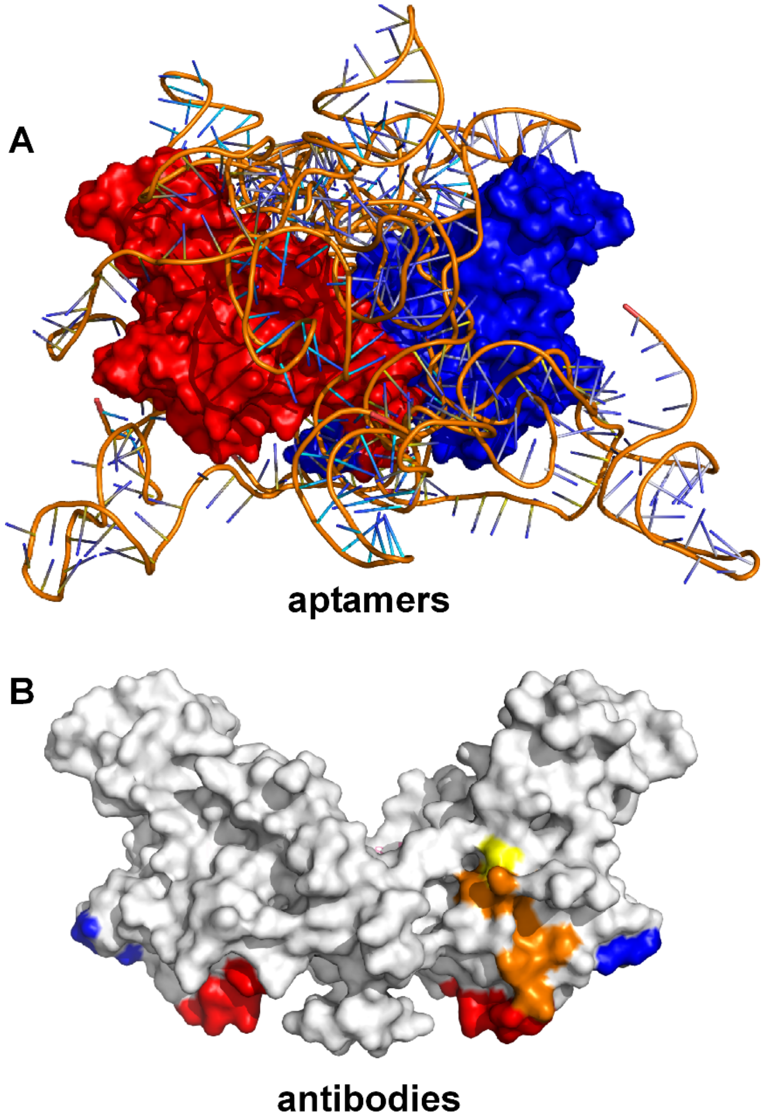

Legend: A) An overlaid view of the top five docking results for oligonucleotide 6011 docking to EBOV sGP (PDB ID 5KEM). EBOV sGP is colored to distinguish between chain A (red) and chain F (blue). The EBOV sGP (PDB ID 5KEM) [15; 16] and EBOV GP (PDB ID 5KEN) [17; 16] were obtained from the RCSB Protein Data Bank site [18]. The Fab domains were removed from the structures. The secondary structures of aptamers were predicted using UNAfold webserver (http://www.unafold.org/) [12] with the following conditions: 137 mM Na^+, 5 mM Mg^{++}, 24 °C. The structures with the minimum free energy were chosen. The 3dRNA/DNA web server (http://biophy.hust.edu.cn/new/3dRNA) [13; 14] was used to predict the aptamer 3D structures (Fig. S4). The HDOCK webserver (http://hdock.phys.hust.edu.cn) [19; 20] was used to dock the EBOV sGP to the aptamers. PyMOL 2.4.4 [21] was used for visualization. **B)** The positions on sGP bound by the antibodies tested in this work are shown in red and orange (α-81-90 rabbit antiserum), yellow and orange (21D10) [22], and blue (FVM-04 and FVM-09) [23]. Note that the primary epitope for FVM-09 is GEWAF (residues 286–290 that are in GP1, but not in sGP) [23].

Figure S5. Overlaid models for aptamer docking to sGP compared with antibody epitopes.

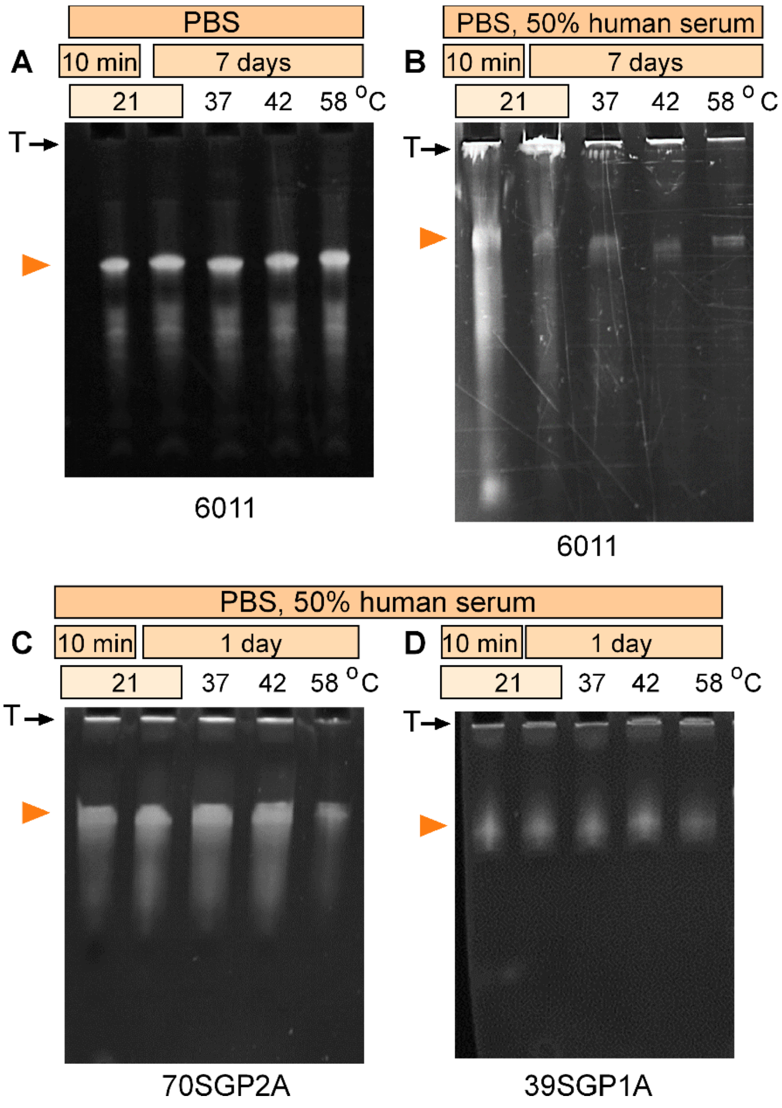

Legend: Oligonucleotides were incubated under the conditions shown in the figure then resolved by Urea PAGE. **A, B**) Ten μM oligonucleotide 6011, **C**) Sixteen μM 70SGP2A, **D**) Eight μM 39SGP1A. T= top of the running gel and bottom of the wells. The oligonucleotide positions in the gels are identified by the orange arrow heads.

Figure S6. Stability of oligonucleotide 6011 at high temperatures and in serum.

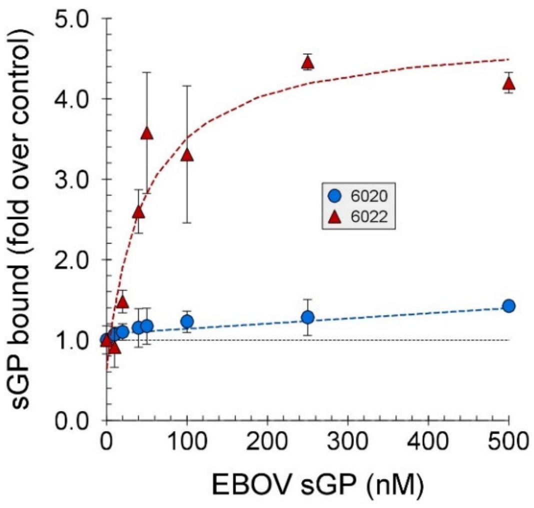

Figure S7. Binding isotherms for oligonucleotides 6020 and 6022.

Legend: The binding isotherms of radiolabeled oligonucleotides 6020 and 6022 were determined using dot-blot and single filter capture assays each performed with duplicate samples. The results (sGP bound) are expressed as cpm bound divided by the control determined in the absence of sGP. The estimated Kd for 6022 was 23 ± 16 nM.

.

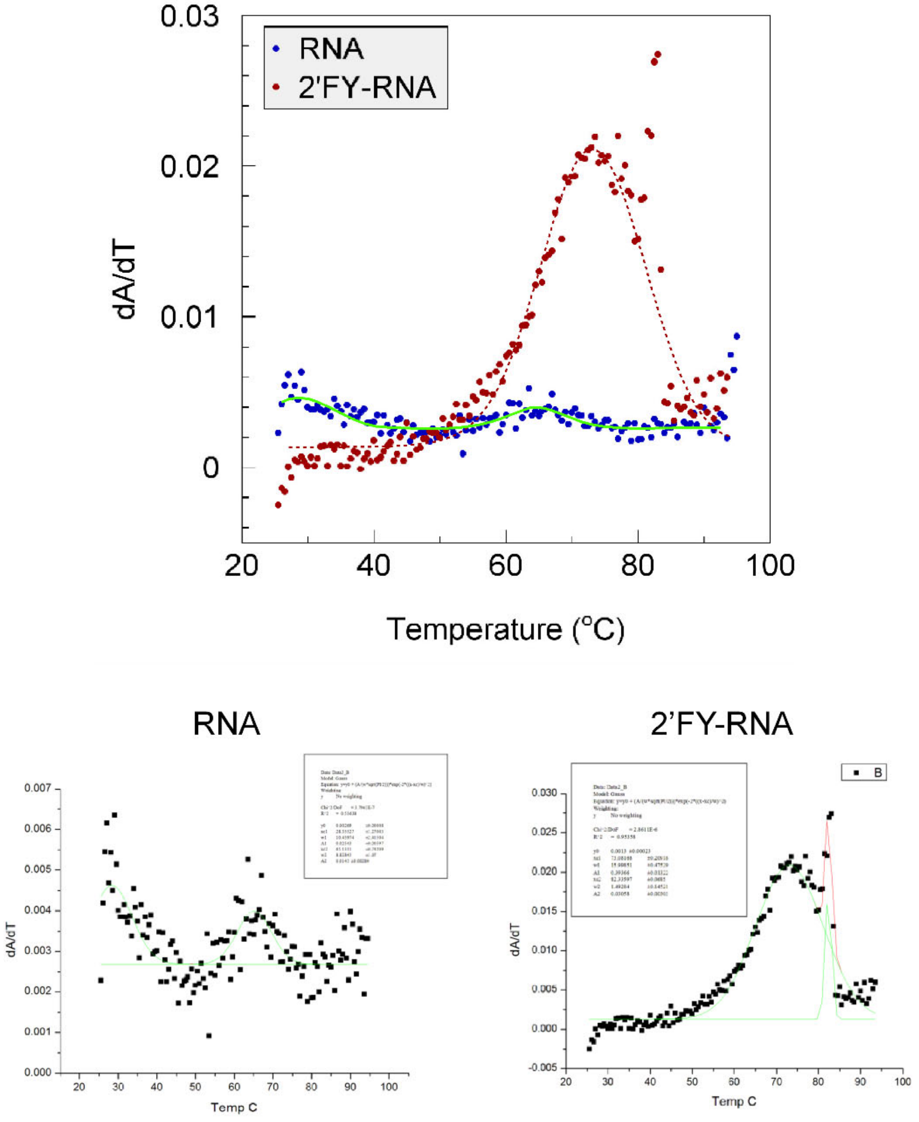

Legend: 39SGP1A was transcribed as RNA or 2’FY-RNA. Melting curves were performed with 2 μM of each oligonucleotide in 100 mM Tris·Cl, pH 7.4 using a Cary100 spectrophotometer and measuring absorbance at 260 nm. The temperature gradient ranged from 25 °C to 95 °C and in the reverse (95 °C to 25 °C) with steps of 0.5 °C/min. Each temperature was held for 1 min. The first derivative of the results (dA/dT) were calculated and fit to a Gaussian function using Origin7 Pro software. **Top:** combined profiles of RNA and 2’FY-RNA melt curves. **Bottom:** showing the fit lines for each titration.

Figure S8. Thermal stability of 39SGP1A as RNA and 2’FY-RNA.

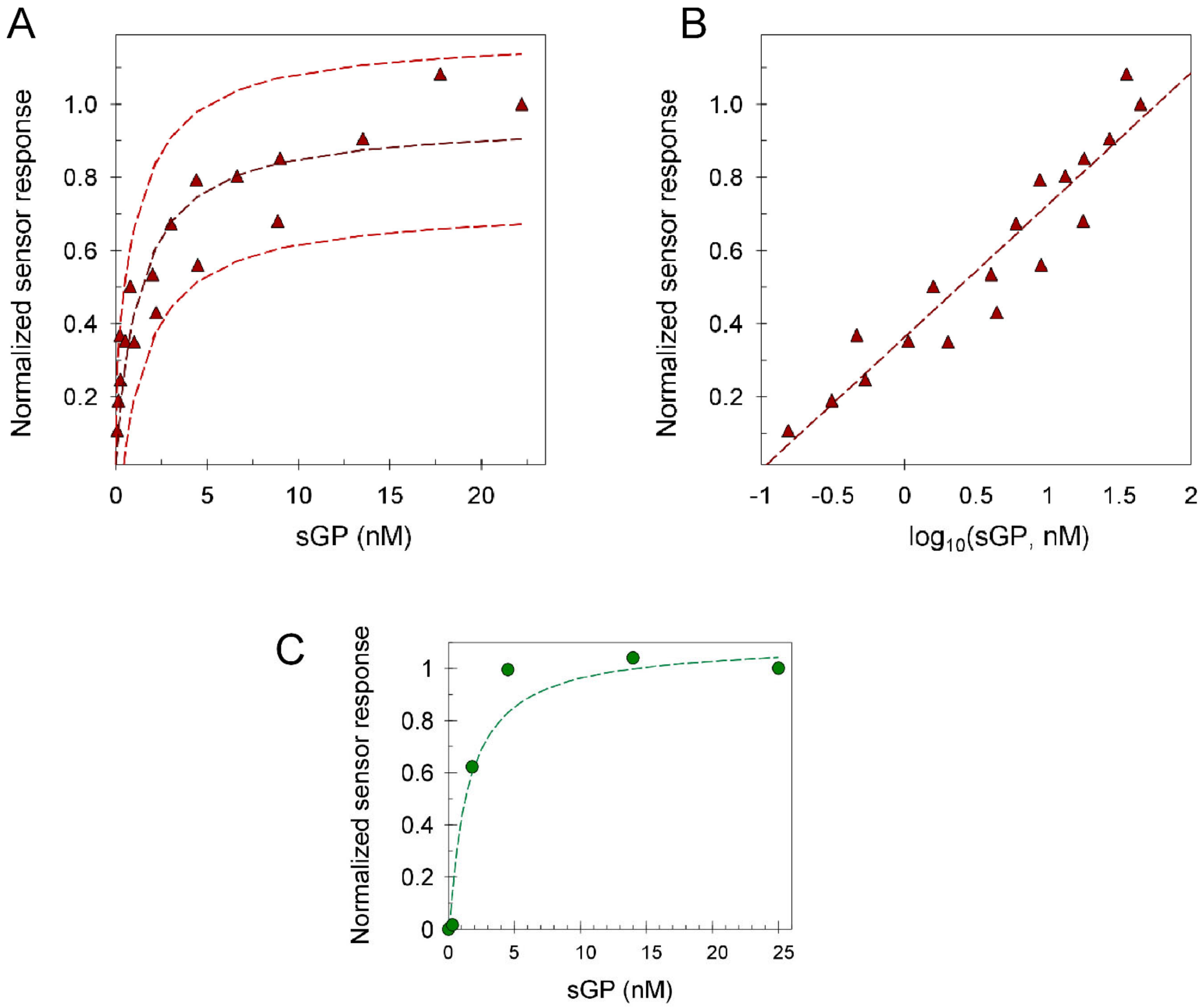

Legend: A) The standard curve shown in Figure 7B, **B)** The same data as in A, but plotted on a semi-log lot to show the linear range as far as we have tested it to 44 nM. Linear fit equation: S = 0.3421*$\log_{10}$[sGP in nM] + 0.3345 ; R^2 = 0.8921. **C)** The data used to create the standard curve for sGP in monkey serum that is shown in Fig. 7C with the fitted binding isotherm shown as a dashed line.

Figure S9. Data for standard curves for EIS analysis.

EIS scans for Fig. 7B

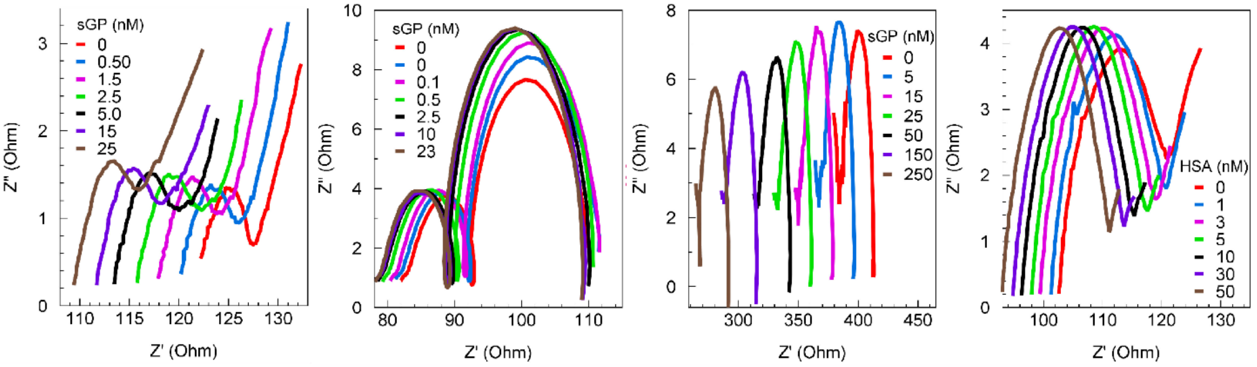

EIS scans for Fig. 7C

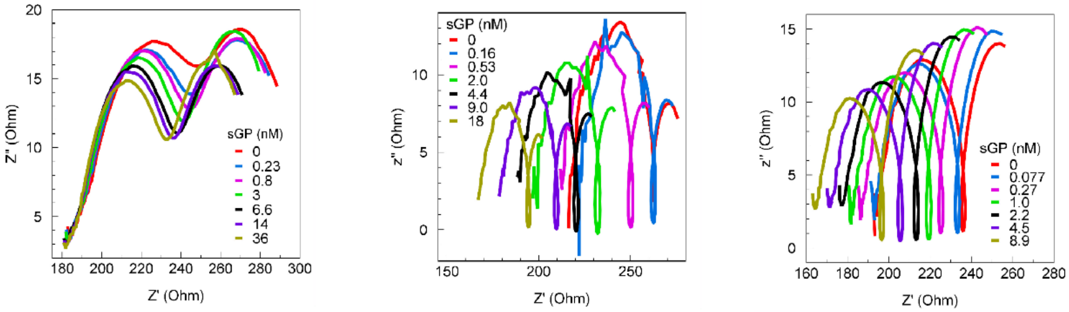

EIS scans for Fig. 7D

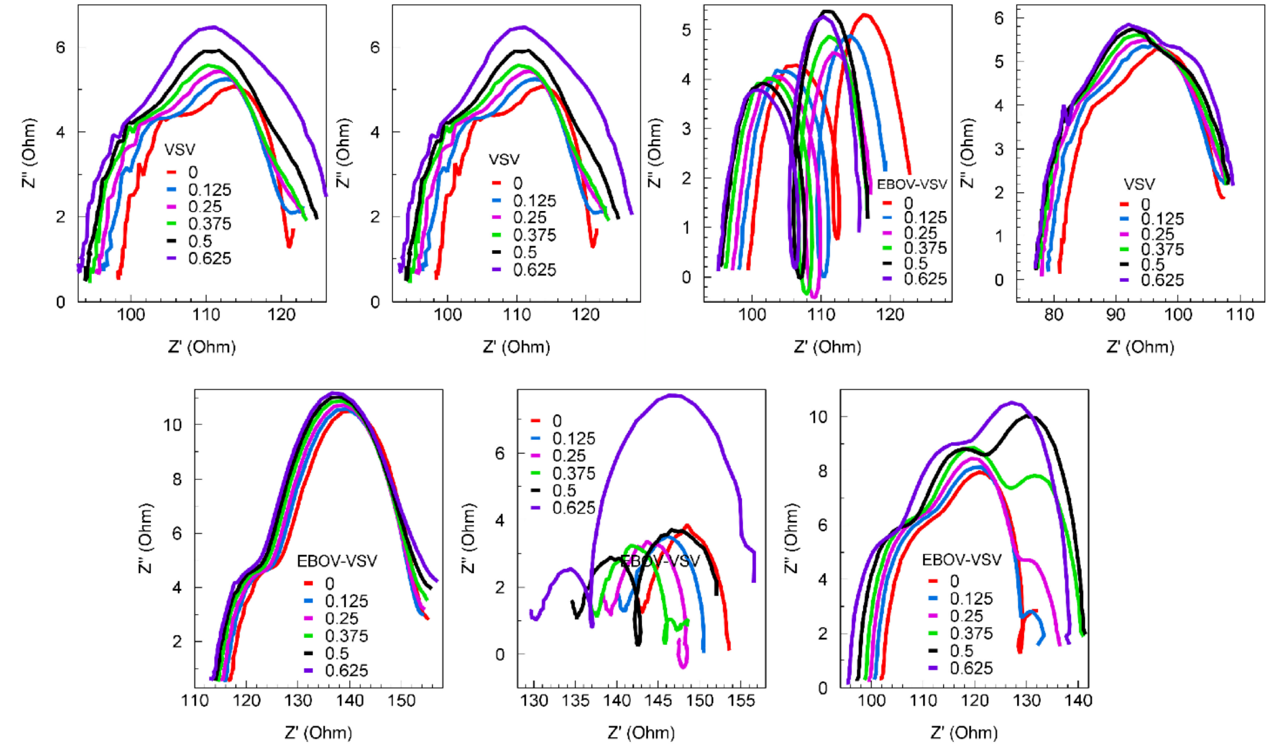

Legend: Nyquist plots for data contributing to the plots in Figure 7B-C. **Figure S10.** Electrochemical sensing Nyquist plots for Figure 7.

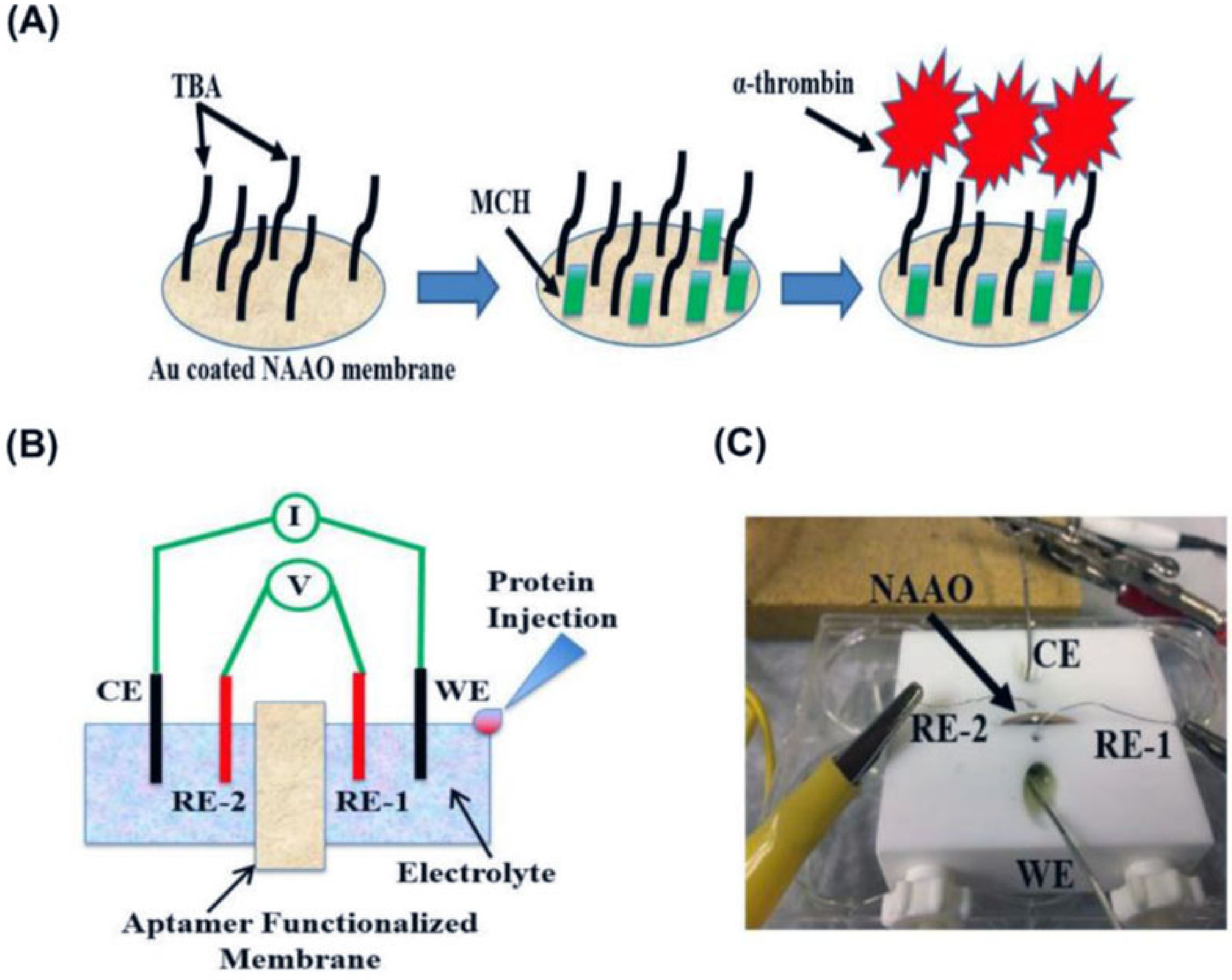

Legend: (A) Schematic showing membrane immobilization steps **(B)** The four-electrode scheme **(C)** the Teflon cell as used in the electrochemical experiments with the NAAO membrane and the four-electrode set-up. Figure 1 from [24] with permission.

Figure S11. Schematic of electrochemical sensor.

Citations

1. Shubham, S., Hoinka, J., Banerjee, S., Swanson, E., Dillard, J.A., Lennemann, N.J., et al. "A 2′ FY-RNA Motif Defines an Aptamer for Ebolavirus Secreted Protein." *Scientific Reports* 8(1) (2018): 12373. https://www.doi.org/10.1038/s41598-018-30590-8
2. Hong, S.-L., Xiang, M.-Q., Tang, M., Pang, D.-W., and Zhang, Z.-L. "Ebola Virus Aptamers: From Highly Efficient Selection to Application on Magnetism-controlled Chips." *Analytical Chemistry* 91(5) (2019): 3367-3373. https://www.doi.org/10.1021/acs.analchem.8b04623
3. Duan, D., Fan, K., Zhang, D., Tan, S., Liang, M., Liu, Y., et al. "Nanozyme-strip for rapid local diagnosis of Ebola." *Biosensors and Bioelectronics* 74(2015): 134-141. https://www.doi.org/10.1016/j.bios.2015.05.025
4. Yen, C.-W., de Puig, H., Tam, J.O., Gómez-Márquez, J., Bosch, I., Hamad-Schifferli, K., et al. "Multicolored silver nanoparticles for multiplexed disease diagnostics: distinguishing dengue, yellow fever, and Ebola viruses." *Lab on a Chip* 15(7) (2015): 1638-1641. https://www.doi.org/10.1039/C5LC00055F
5. Daaboul, G.G., Lopez, C.A., Chinnala, J., Goldberg, B.B., Connor, J.H., and Ünlü, M.S. "Digital Sensing and Sizing of Vesicular Stomatitis Virus Pseudotypes in Complex Media: A Model for Ebola and Marburg Detection." *ACS Nano* 8(6) (2014): 6047-6055. https://www.doi.org/10.1021/nn501312q
6. Yanik, A.A., Huang, M., Kamohara, O., Artar, A., Geisbert, T.W., Connor, J.H., et al. "An Optofluidic Nanoplasmonic Biosensor for Direct Detection of Live Viruses from Biological Media." *Nano Letters* 10(12) (2010): 4962-4969. https://www.doi.org/10.1021/nl103025u
7. Cai, H., Parks, J.W., Wall, T.A., Stott, M.A., Stambaugh, A., Alfson, K., et al. "Optofluidic analysis system for amplification-free, direct detection of Ebola infection." *Scientific Reports* 5(2015): 14494. https://www.doi.org/10.1038/srep14494
8. Chen, Y., Ren, R., Pu, H., Guo, X., Chang, J., Zhou, G., et al. "Field-Effect Transistor Biosensor for Rapid Detection of Ebola Antigen." *Scientific Reports* 7(1) (2017): 10974. https://www.doi.org/10.1038/s41598-017-11387-7
9. Benzine, J.W., Brown, K.M., Agans, K.N., Godiska, R., Mire, C.E., Gowda, K., et al. "Molecular Diagnostic Field Test for Point-of-Care Detection of Ebola Virus Directly From Blood." *The Journal of Infectious Diseases* 214(Suppl 3) (2016): S234-S242. https://www.doi.org/10.1093/infdis/jiw330
10. Zang, F., Su, Z., Zhou, L., Konduru, K., Kaplan, G., and Chou, S.Y. "Ultrasensitive Ebola Virus Antigen Sensing via 3D Nanoantenna Arrays." *Advanced Materials* 31(30) (2019): 1902331. https://www.doi.org/https://doi.org/10.1002/adma.201902331
11. Jin, X., Zhang, H., Li, Y.-T., Xiao, M.-M., Zhang, Z.-L., Pang, D.-W., et al. "A field effect transistor modified with reduced graphene oxide for immunodetection of Ebola virus." *Microchimica Acta* 186(4) (2019): 223. https://www.doi.org/10.1007/s00604-019-3256-5
12. Markham, N.R., and Zuker, M. (2008). "UNAFold," in *Bioinformatics: Structure, Function and Applications,* ed. J.M. Keith. (Totowa, NJ: Humana Press), 3-31.
13. Wang, J., Wang, J., Huang, Y., and Xiao, Y. "3dRNA v2.0: An Updated Web Server for RNA 3D Structure Prediction." *Int J Mol Sci* 20(17) (2019). https://www.doi.org/10.3390/ijms20174116
14. Zhang, Y., Xiong, Y., and Xiao, Y. "3dDNA: A Computational Method of Building DNA 3D Structures." *Molecules* 27(18) (2022): 5936. https://www.doi.org/10.3390/molecules27185936
15. PDB *http://dx.doi.org/10.2210/pdb5kem/pdb* [Online]. [Accessed 10/6/2022].
16. Pallesen, J., Murin, C.D., de Val, N., Cottrell, C.A., Hastie, K.M., Turner, H.L., et al. "Structures of Ebola virus GP and sGP in complex with therapeutic antibodies." *Nature Microbiology* 1(9) (2016): 16128. https://www.doi.org/10.1038/nmicrobiol.2016.128
17. PDB *http://dx.doi.org/10.2210/pdb5ken/pdb* [Online]. [Accessed 10/20/2022].
18. Berman, H.M., Westbrook, J., Feng, Z., Gilliland, G., Bhat, T.N., Weissig, H., et al. "The Protein Data Bank." *Nucleic Acids Res* 28(1) (2000): 235-242. https://www.doi.org/10.1093/nar/28.1.235

19. Huang, S.Y., and Zou, X. "A knowledge-based scoring function for protein-RNA interactions derived from a statistical mechanics-based iterative method." *Nucleic Acids Res* 42(7) (2014): e55. https://www.doi.org/10.1093/nar/gku077
20. Yan, Y., Zhang, D., Zhou, P., Li, B., and Huang, S.Y. "HDOCK: a web server for protein-protein and protein-DNA/RNA docking based on a hybrid strategy." *Nucleic Acids Res* 45(W1) (2017): W365-W373. https://www.doi.org/10.1093/nar/gkx407
21. The PyMol Molecular Graphics System, V.S., LLC. Available: https://pymol.org/2/ [Accessed 9/16/2022 to 12/29/2022].
22. Holtsberg, F.W., Shulenin, S., Vu, H., Howell, K.A., Patel, S.J., Gunn, B., et al. "Pan-ebolavirus and Pan-filovirus Mouse Monoclonal Antibodies: Protection against Ebola and Sudan Viruses." *J Virol* 90(1) (2016): 266-278. https://www.doi.org/10.1128/jvi.02171-15
23. Keck, Z.-Y., Enterlein, S.G., Howell, K.A., Vu, H., Shulenin, S., Warfield, K.L., et al. "Macaque Monoclonal Antibodies Targeting Novel Conserved Epitopes within Filovirus Glycoprotein." *Journal of Virology* 90(1) (2016): 279. https://www.doi.org/10.1128/JVI.02172-15
24. Gosai, A., Yeah, B.S.H., Nilsen-Hamilton, M., and Shrotriya, P. "Label Free Thrombin Detection in Presence of High Concentration of Albumin Using an Aptamer-Functionalized Nanoporous Membrane." *Biosensors and Bioelectronics* 126(2019): 88-95. https://www.doi.org/10.1016/j.bios.2018.10.010
